# Positron annihilation spectroscopy of vacancy-related defects in CdTe:Cl and CdZnTe:Ge at different stoichiometry deviations

**DOI:** 10.1038/srep20641

**Published:** 2016-02-10

**Authors:** L. Šedivý, J. Čížek, E. Belas, R. Grill, O. Melikhova

**Affiliations:** 1 Institute of Physics, Charles University in Prague, Ke Karlovu 5, CZ-121 16, Prague 2, Czech Republic; 2 Department of Low-Temperature Physics, Charles University in Prague, V Holešovičkách 2, CZ-180 00, Prague 8, Czech Republic

## Abstract

Positron annihilation spectroscopy (PAS) was used to examine the effect of defined Cd-rich and Te-rich annealing on point defects in Cl-doped CdTe and Ge-doped CdZnTe semi-insulating single crystals. The as-grown crystals contain open-volume defects connected with Cd vacancies 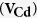. It was found that the Cd vacancies agglomerate into clusters coupled with Cl in CdTe:Cl, and in CdZnTe:Ge they are coupled with Ge donors. While annealing in Cd pressure reduces of the 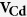 density, subsequent annealing in Te pressure restores 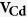. The CdTe:Cl contains negatively-charged shallow traps interpreted as Rydberg states of 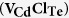 A-centres and representing the major positron trapping sites at low temperature. Positrons confined in the shallow traps exhibit lifetime, which is shorter than the CdTe bulk lifetime. Interpretation of the PAS data was successfully combined with electrical resistivity, Hall effect measurements and chemical analysis, and allowed us to determine the principal point defect densities.

Cadmium telluride (CdTe) and Cadmium zinc telluride (CdZnTe) are important materials for multiple applications in solar cells^1^,^2^,^3^, X-ray and gamma-ray room-temperature detectors^4^,^5^, electro-optic modulators^6^, and substrates for CdHgTe infra-red detector epitaxy^7^. In case of room-temperature semiconductor detectors these compounds overcome convention materials (Si, Ge, GaAs) by optimum conjunction of principal parameters - large atomic number of elements, large enough band gap energy, convenient electron mobility and attainable high resistivity and low enough trap density - necessary for successful detector manufacture^8^.

In spite of the high interest and expectation on CdTe/CdZnTe-detectors, till nowadays they have been hardly commercialized due to high expenses for fabrication high quality crystals. A CdTe crystal with a high resistivity 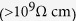 is required for a good detector in order to reduce the dark current. Moreover a large mobility-lifetime product is desired to ensure that carriers generated by radiation in the whole detector volume can be collected by electrodes. Lattice defects form energy levels in the band gap and affect thereby the resistivity and the mobility lifetime product^9^. CdTe can be alloyed with Zn forming CdZnTe ternary compound. Alloying of CdTe with Zn strengthens the lattice and increases the band gap and thereby also maximum achievable resistivity^10^.

The electric properties are affected by lattice defects forming energy levels in the band gap^9^. The determination of the properties of native defects and their complexes is particularly important to understand their role in crystal growth and compensating of extrinsic defects^4^. In spite of extensive investigations the defects attached to distinct levels have in most cases not been completely identified. High purity CdTe/CdZnTe crystals are usually grown under Te-rich conditions because the Cd pressure required for Cd-rich conditions is as high as several atmospheres^11^. The CdTe/CdZnTe crystals grown under Te-rich conditions are typically characterized by low resistivity and p-type conductivity^4^ due to Cd vacancies 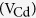 which are divalent acceptors with two ionization levels 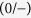 and 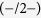 located below the mid-gap level^4^,^12^. To achieve high resistivity 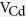 acceptors and residual impurities must be electrically compensated usually by doping using group III (Al, In, Ga) or group VIII (Cl) donors^4^,^12^.

Positron annihilation spectroscopy (PAS) including Positron lifetime (LT) and Coincidence Doppler broadening (CDB) spectroscopy was usually used for the study of the structure of point defects in CdTe/CdZnTe in the past^13^,^14^,^15^,^16^,^17^,^18^, but the link between defects and controlled annealing treatment has never been studied before.

This work reports on an investigation of the influence of defined annealing in Cd or Te vapour on the defect structure of Cl-doped CdTe and Ge-doped 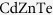 semi-insulating single crystals. The defect structure is characterized by PAS before and after each annealing step. The results are compared with galvanomagnetic Hall effect measurements in the Van der Pauw configuration^19^.

The paper is organized as follows. In Section Experimental we introduce samples, show the impurity content and summarize technical details of experiments. In Section Theory, we introduce methods for lifetime calculations and discuss their limits. Section Results and Discussion presents galvanomagnetic, positron back-diffusion measurements and theoretical calculations of lifetimes of positrons captured in different point defects. The subsection Lifetime spectroscopy reports the results of temperature dependent positron lifetime spectroscopy and their analysis based on three-state simple trapping model. This subsection is divided to the three parts describing for the undoped CdTe, Cl-doped CdTe and Ge-doped CdZnTe. The next subsection contains the results of CDB spectroscopy and their interpretation.

## Experimental

The Cl-doped CdTe (CdTe:Cl) and Ge-doped 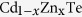, 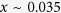 (CdZnTe:Ge) single crystals were grown by the vertical gradient freeze method^20^ (using 6N purity source elements). The annealing was done in the two zone furnace at 700 °C while the Cd or Te source temperature was 600 °C, which corresponds to Cd or Te pressure of 111 or 7.7 mbar, respectively. After annealing, the samples were cooled down at a cooling rate of 1 °C min^−1^. Nominally undoped CdTe (u-CdTe) single crystal grown by the vertical gradient freeze method at fairly increased Cd overpressure 1.6 atm was used as a reference sample.

LT measurements were carried out using a digital spectrometer^21^ with time resolution of 145 ps. ^22^Na activity of 1 MBq deposited on a 2 *μ*m thick Ti foil was used as a positron source. The LT measurements were performed at 295 K (room temperature - RT) and at 123 K (low temperature), respectively. In both temperatures typically, 10^7^ positron annihilation events were collected in LT spectra, which were subsequently decomposed into exponential components. The contribution of positron annihilations in the source spot and in the covering foils were always subtracted. In addition, temperature-dependent LT measurements were carried out in the temperature range of 123–295 K at a heating rate of ~1 K hour^−1^. Here the statistics of 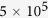 events was collected at each temperature step.

CDB spectroscopy^22^ was employed for characterization of local chemical environment of vacancies. The CDB studies were performed at RT using the same positron source as in the LT measurements. A digital CDB spectrometer^23^ equipped with two HPGe detectors and characterized by the energy resolution of 0.9 keV at the annihilation line and the peak-to -background ratio 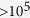 was employed for the CDB studies. At least 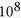 annihilations were collected in each two-dimensional CDB spectrum. Subsequently the CDB spectra were reduced into one-dimensional cuts representing the resolution function of the spectrometer and the Doppler broadened annihilation peak. Normalized Doppler broadened peaks were divided by the normalized peak for the u-CdTe reference sample. Hence, in this paper the CDB results are presented as ratio curves with respect to u-CdTe.

Positron back diffusion measurement was performed on a continuous magnetically guided slow positron beam with energy of incident positrons adjustable in the range from 0.05 to 30 keV. Doppler broadening of the annihilation photopeak was measured by a HPGe detector with the relative efficiency of 35% and the energy resolution of 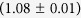 keV at 511 KeV. Shape of Doppler broadened annihilation photopeak was characterized using the *S* (sharpness) parameter^24^. The dependence of of the *S* parameter on positron energy was fitted using the VEPFIT code^25^.

Glow Discharge Mass Spectrometry (GDMS) was used for the chemical analysis. The concentrations of elements exceeding 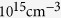 for u-CdTe, CdTe:Cl and CdZnTe:Ge presented in Table 1 in units 10^15^ cm^−3^. A 30% error of GDMS analysis was declared.

## Theory

Positron properties were calculated using density functional theory (DFT) within so-called standard scheme^26^. In this approximation positron density is assumed to be everywhere vanishingly small and not affecting the bulk electron structure. At first electron density 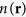 in the material is solved without the positron. Subsequently, the effective potential for positron is constructed as


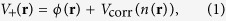


where 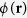 is the Coulomb potential produced by the charge distribution of electrons and nuclei and 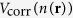 is the zero positron density limit of the electron-positron correlation potential^26^.

Positron wave functions 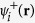 were calculated by solution of a single particle Schrödinger equation


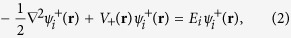


where 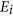 is the energy eigenvalue for *i*-th positron state. In the present calculations we considered the positron ground state only.

The positron annihilation rate (i.e. the inverse of positron lifetime) is determined using the expression


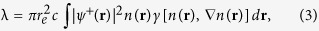


where 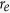 is the classical electron radius, *c* is the speed of light, and *γ* denotes the electron enhancement factor describing the pileup of electrons at the positron site^26^.

The electron-positrons correlation, i.e. the correlation potential 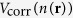 and the enhancement factor *γ*, were treated by two approaches:

- local density approximation (LDA) utilizing the parametrization by Boroński and Nieminen^27^ and taking into account incomplete positron screening^28^ using a high frequency dielectric constant of 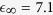
29 and
- generalized gradient approach (GGA) within the approach introduced by Barbiellini *et al*.^30^.

The electron density 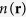 was constructed by superposition of atomic electronic densities calculated by a relativistic atomic code^31^. This approach called atomic superposition (ATSUP)^32^ neglects the charge transfer, but it is computationally feasible and can be used even for very large supercells retaining full 3-D geometry of the problem. In the following text the approach employing LDA and GGA scheme with superimposed electron density is denoted ATSUP-LDA and ATSUP-GGA, respectively.

The ATSUP-LDA and ATSUP-GGA calculations of positron parameters were performed on 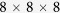 supercells containing 2048 Cd and 2048 Te atoms. Defects were modelled by removing the corresponding number of atoms from the supercell. Integration over the Brillouin-zone described in ref. 33 was used in calculations of positron parameters for defects.

It is well known that LDA overestimates positron annihilation rates especially with *d* electrons^30^ and the lifetimes calculated within the LDA approach are often shorter than the values measured in experiment. This shortcoming is to some extend compensated when LDA approach is used with electron density constructed by ATSUP^32^,^34^. On the other hand, the GGA approach when used with ATSUP electron density gives often lifetimes which are longer than the experimental values^35^. Hence, the lifetimes calculated using the LDA and the GGA approach can be considered as a lower and an upper bound of the interval where the actual lifetime falls. The GGA scheme is more sensitive to details of electronic structure than LDA^30^ and the lifetimes calculated using the GGA approach are it the best agreement with experiment when used with a self-consistent electron density. For these reasons we performed also self-consistent electron density calculations for selected positron states. The self consistent valence electron density was calculated by the plane wave code VASP (Vienna ab-inito simulation package)^36^,^37^ using projector augmented wave (PAW) potentials^38^. The 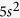, 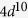 Cd electrons and 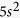, 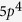 Te electrons were considered as valence electrons in the VASP calculations. The calculations were performed using 216 atoms based supercells and 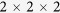 *k*-point grids generated using the Monkhorst-Pack scheme^39^. The wave functions were expanded in a plane wave basis with the cut-of energy of 277 eV. The calculated CdTe lattice constant *a* = 6.60 Å is in a reasonable agreement with the experimental value of 6.48 Å. Ionic relaxations were not considered at the present stage of calculations. In construction of the positron potential the frozen core electron orbitals were added to the self-consistent valence electron density calculated by VASP. In the following text this approach is denoted VASP-GGA.

The momentum distribution of annihilating pairs was calculated employing the ATSUP-based approach described in refs 40 and 41. The electron-positron correlations were treated within the GGA approach. The contribution 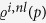 from the *i–*th atom and a shell characterized by principal (*n*) and orbital (*l*) quantum numbers is calculated by the formula


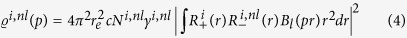


where 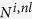 denotes the number of electrons in the 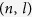 shell, 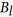 is the spherical Bessel function and 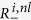 and 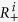 denotes the electron and positron radial wave functions. The symbol 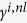 stands for the state-dependent positron enhancement factor^41^. The momentum distribution of the annihilating electron-positron pairs is obtained by summing the partial contributions 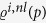 over all occupied atomic sites and corresponding electron shells. Core electrons localized in atomic shells are practically not affected by crystal bonding. Hence, the ATSUP-based approach describes well the high momentum part of the momentum distribution where the contribution of positrons annihilated by core electrons dominates. But since it neglects the charge transfer it fails to describe properly the low momentum part of the momentum distribution defined by positrons annihilated by low momentum valence electrons.

In order to mimic the effect of the finite resolution of the experimental setup, the theoretical momentum distribution curves were convoluted with a Gaussian with FWHM of 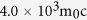. To highlight the high momentum part of momentum distributions where the contribution of core electrons dominates the calculated momentum distributions are presented as ratio curves related to a perfect CdTe crystal.

## Results and Discussion

### Galvanomagnetic measurements

The galvanomagnetic properties of as-grown u-CdTe, CdTe:Cl and CdZnTe:Ge and annealed CdTe:Cl and CdZnTe:Ge samples are summarized in Table 2. The properties of u-CdTe accord with its growth at Cd overpressure, where the *V*_*Cd*_-related defects are suppressed and the electron density is afforded by residual noncompensated donors. The as-grown CdTe:Cl and CdZnTe:Ge samples exhibit rather high resistivity, which is typical for the growth of Te-rich materials, where excess donors dominating above shallow acceptors are compensated by V_Cd_^8^,^42^.

Annealing in Cd vapour leads to a decrease in resistivity for both samples. CdTe:Cl exhibited *n*-type conductivity due to the suppressed V_Cd_ and dominating shallow donors Cl_Te_. The free carrier concentration of ~10^17^ cm^−3^ therefore gives a lower limit for the concentration of Cl dopants in the sample^43^. Cd vapour annealing of CdZnTe:Ge yield *p*-type conductivity contrary to CdTe:Cl. Such a behaviour is well known in annealed undoped or weakly doped CdTe/CdZnTe^44^, where acceptor-like impurities are dominant after annealing. In our CdZnTe:Ge, the extrinsic shallow donor ((B) + (Al)) and shallow acceptor ((P) + (Au) + (Cu)) densities are similar, and weak excess acceptor contamination is assumed. Subsequent annealing in Te vapour produced *p*-type conductivity in both samples. This is in agreement with expected V_Cd_ formation.

### Calculated positron lifetimes

Positron lifetimes, calculated using ATSUP-LDA, ATSUP-GGA and VASP-GGA approaches for various positron states in CdTe are listed in Table 3. Theoretical positron lifetimes available in literature are shown in Table 3 as well. The positron lifetimes calculated using the ATSUP-LDA and ATSUP-GGA schemes in the present work are in an excellent agreement with the lifetimes calculated in refs 15,45. One can see in Table 3 that positron lifetimes calculated using various approaches for the electron-positron correlation, i.e. the enhancement factor and the electron-positron correlation potential, differ. The positron lifetimes calculated using the LDA approach are always lower than those obtained by the GGA scheme. Moreover, calculated positron lifetimes vary depending whether superimposed or self-consistent electron density was used in the calculation. The bulk positron lifetime for CdTe calculated using the ATSUP-LDA approach is 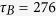 ps, while the ATSUP-GGA scheme yielded higher bulk lifetime 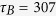 ps. For comparison experimental lifetimes reported in literature for CdTe are listed in Table 4. The experimental bulk positron lifetimes for CdTe reported in literature fall into the range from 280 to 291 ps^13^,^14^,^15^,^16^,^18^,^46^,^47^. The VASP-GGA scheme which can be considered as the most precise approach used here yielded the CdTe bulk positron lifetime 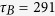 ps which is comparable with the experimental values.

In order to compare positron lifetimes for various defects with the experimental values we use ratios 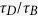 of the calculated lifetime 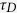 to the bulk lifetime 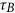 calculated by the same approach. These ratios are listed in Table 5. The differences between various theoretical schemes are to a large extend cancelled using 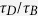 ratios and the 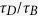 values exhibit relatively low sensitivity to the approach used for electron-positron correlation^30^,^33^.

**Figure 1:**
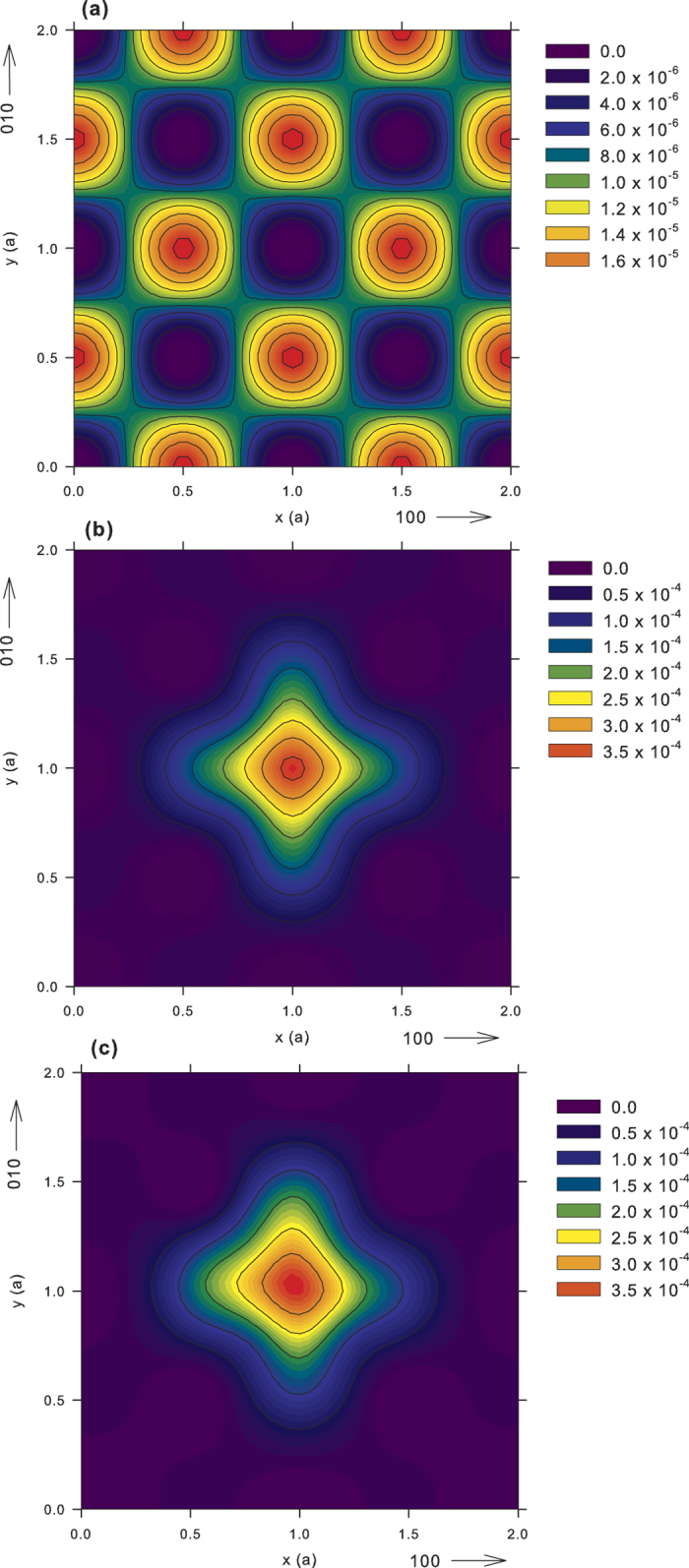
Positron density in the (001) plane calculated using the ATSUP-GGA approach (**a**) perfect CdTe crystal, (**b**) CdTe crystal containing V_*Cd*_ in 1, 1, 0 position, (**c**) CdTe crystal containing A_C_ defect consisting of V_Cd_ and Cl_Te_ located in 1, 1, 0 and 0.25, 1.75, 0.25 position, respectively. The co-ordinates are expressed in the units of the CdTe lattice constant *a*. Positron density is given in the atomic units.

While 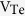 is believed to be positively charged in CdTe, 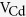 is either neutral 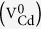 or negatively charged 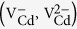 depending on the Fermi level position^12^. Hence, positrons are repelled by 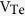 while 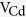 represent trapping sites for positrons. This is illustrated in Fig. 1 presenting the positron density in the (001) plane calculated by the ATSUP-GGA approach. Figure 1a shows the positron density in a perfect CdTe crystal where positron is de-localized in the lattice. The positron density calculated for a CdTe crystal containing 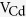 is plotted in Fig. 1b. Obviously the positron is localized in 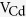. Experimental evidence for positron trapping in 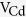 has been reported by many authors^14^,^15^,^16^,^18^,^46^. The lifetimes of positrons trapped 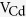 determined experimentally fall into the range from 320 to 325 ps which corresponds to 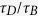 falling into the interval 1.11–1.15, see Table 4. The ATSUP-LDA and ATSUP-GGA calculations for positrons trapped at 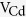 resulted in 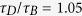, which is remarkably lower than the experimental values. On the other hand, the VASP-GGA approach yielded 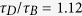 which is close to the values measured in experiment. This indicates that charge transfer which was neglected in ATSUP calculations leads to a slight decrease of electron density in 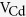 and consequently an increase of the lifetime of trapped positrons.

Positron lifetime was calculated also for 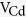 associated with Te anti-site 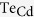 and with various impurities. One can see in Table 3 that 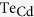 causes shortening of positron lifetime. A 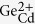 donor associated with 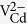 acceptor forms 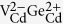 complex which is electrically neutral. Theoretical calculations revealed that positrons trapped at 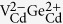 complexes exhibit practically the same lifetime as 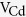. Hence, these two kinds of defects cannot be distinguished by measurement of positron lifetime.

In Cl doped CdTe 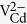 may couple with 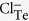 shallow donors forming negatively charged 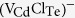 complexes called A-centres 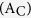 or neutral 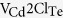 complexes. Results in Table 3 indicate that replacement of Te nearest neighbours of 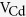 by Cl atoms increases the lifetime of trapped positron. This effect is caused by smaller size of Cl atoms compared to Te ones. Hence replacement of a Te nearest neighbour of 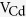 by a Cl impurity increases the open volume of vacancy. This is illustrated in Fig. 1c which presents calculated positron density in the (001) plane for a positron trapped at A_C_ defect. One can see in the figure that positron density in A_C_ becomes asymmetric since it expanded towards the Cl nearest neighbour.

### Lifetime spectroscopy

**Figure 2:**
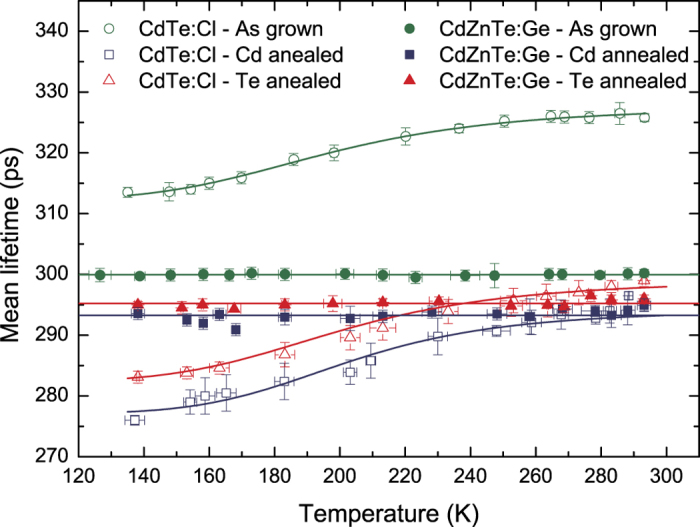
Temperature dependence of the mean positron lifetime for CdTe:Cl and CdZnTe:Ge. The lines represent the fit using 3-STM.

The results of LT measurements, i.e. lifetimes 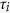 and relative intensities 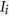 resolved in LT spectra for u-CdTe, CdTe:Cl and CdZnTe:Ge samples, are summarized in Table 6 for temperatures 295 and 123 K, respectively. The temperature dependences of the mean positron lifetime 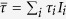 are presented in Fig. 2.

### Undoped CdTe

The u-CdTe crystal exhibits a single component LT spectrum (except of the source contribution) with lifetime of ~290 ps, which fits in the CdTe bulk lifetimes reported in literature, see Table 4. Hence, the concentration of positron traps in the u-CdTe crystal is very low (below the sensitivity threshold of LT spectroscopy) and virtually all positrons are annihilated in the de-localized state. Low temperature LT measurements of the u-CdTe crystal yielded practically the same results as the measurement at RT.

**Figure 3:**
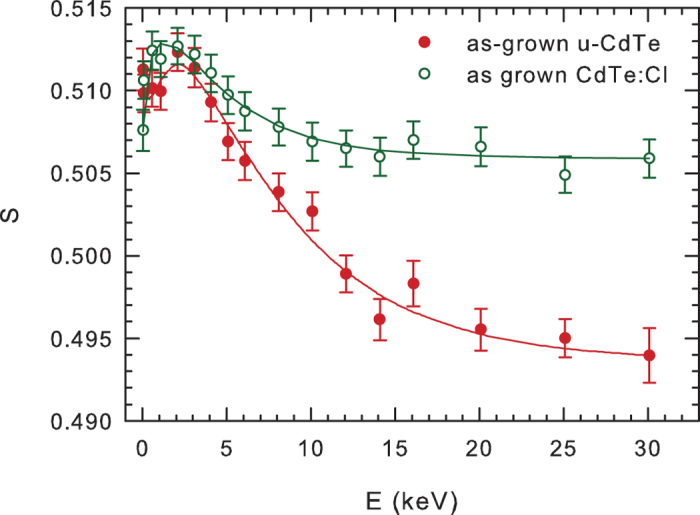
The dependence of the *S* parameter on the energy *E* of incident positrons for undoped CdTe (full symbols) and CdTe:Cl sample in the as-grown state (open symbols). Solid lines show model curves calculated by VEPFIT.

Results of positron back-diffusion measurement, i.e. dependence of the *S* parameter on the energy *E* of incident positrons in plotted in Fig. 3. The positron penetration depth increases with increasing energy of implanted positrons. Hence, at very low energies virtually all positrons are annihilated at the surface. With increasing energy positrons penetrate deeper into the sample and the fraction of positrons diffusing back to the surface gradually decreases. Finally at high energies virtually all positrons are annihilated in the bulk and the *S* parameter approaches a plateau. One can see in Fig. 3 that the 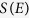 curve exhibits a maximum at energy around 2 keV. This indicates that the sample contains a thin surface layer of native oxides most probably a mixture of 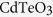, 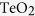 and CdO^48^,^49^. The 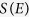 curve for the u-CdTe sample was fitted by VEPFIT^25^ using a two layer model: (i) native oxide layer on the surface and (ii) CdTe bulk region. The model curve calculated by VEPFIT is plotted in Fig. 3 by a solid line and is obviously in good agreement with the experimental points. The thickness of the native oxide layer ≈30 nm was fitted.

The positron diffusion length determined in the CdTe region is 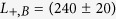 nm. This value is comparable with the positron diffusion lengths in defect-free semiconductors^24^. Hence, the u-CdTe crystal contains very low density of positron traps (except of the thin surface oxide layer) and almost all positrons are annihilated from the free state in accordance with the results of positron lifetime characterization. Assuming that the u-CdTe sample contains no positron traps one can calculate the positron diffusion coefficient for u-CdTe from the expression 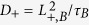, where 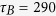 ps is the bulk u-CdTe lifetime. This yields the positron diffusion coefficient for u-CdTe 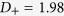 cm^2^s^−1^ which is in very good agreement with the value 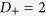 cm^2^s^−1^ reported for CdTe by Neretina *et al*.^50^.

The u-CdTe crystal exhibits very low density of dislocations which does not exceeds 10^9^ m^−2^ as determined by etch pitting technique. We performed an additional independent test whether positrons in u-CdTe crystal are annihilated in the free state and the lifetime of ~290 ps measured in this sample can be indeed considered as the bulk lifetime. The u-CdTe sample was mechanically grinded by F600 *SiC* paste with grain size 9 *μ*m. Plastic deformation caused by grinding introduced dislocations into the sample. The grinded u-CdTe sample exhibits two-component LT spectrum with lifetimes 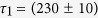 ps and 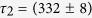 ps and corresponding relative intensities 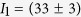
% and 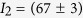
%. Hence, it is clear that grinding introduced defects into the sample. The lifetime 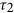 of the second component is comparable with the lifetime of positrons trapped at 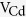 (see next section). This is typical for positrons trapped at dislocations^24^,^51^. Thus, the component with lifetime 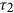 can be attributed to positrons trapped at dislocations introduced by grinding. The lifetime 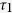 of the first component was shortened with respect to the as-grown u-CdTe sample. This is in accordance with the two-state simple trapping model^24^,^52^. When positrons are trapped at defects then the lifetime 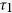 of the free positron component becomes shorter than that in the defect-free material and the quantity


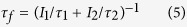


equals to the positron bulk lifetime, i.e. 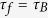
24,52. For the grinded u-CdTe Eq. (5) yielded 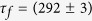 ps which is in an excellent agreement with the lifetime measured in the as-grown u-CdTe. This gives strong evidence that the lifetime of ~290 ps measured in the as-grown u-CdTe comes indeed from annihilations of free positrons. If the lifetime measured in the as-grown u-CdTe would come from positrons trapped at some defects, then introduction of additional defects (here dislocations) by grinding would not cause any shortening of the lifetime 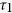.

### CdTe:Cl

The LT spectra of the CdTe:Cl crystal generally consist of up to three components (except of the source contribution) corresponding to the three positron states: (i) free positrons (lifetime *τ*_1_), (ii) positrons trapped in shallow traps (lifetime 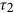, trapping rate 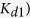, and (iii) positrons trapped in deep traps (lifetime 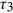, trapping rate 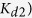. The kinetics of positron trapping for these defects is described by the three-state simple trapping model (3-STM)^24^. The rate equations for such system read^24^


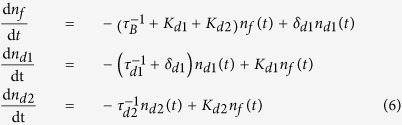


with the initial conditions 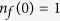 and 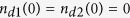. The symbols 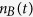, 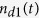 and 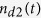 denote the probabilities that a positron is at time *t* de-localized in the free state or trapped in a shallow or deep trap, respectively. 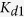 and 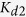 stand for the positron trapping rates for the shallow and the deep traps, respectively, and 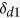 is the de-trapping rate from the shallow traps. The lifetimes of positrons trapped at the shallow and the deep traps, i.e. inverse of the annihilation rates of trapped positrons, are denoted 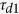 and 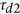, respectively. Solution of the differential equations (6) gives the decay spectrum of positrons


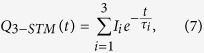


where


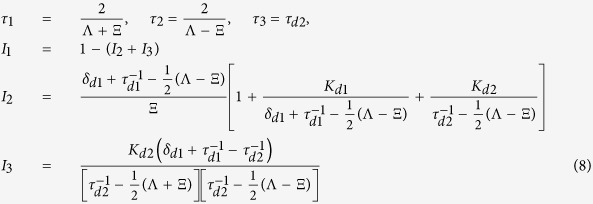


and


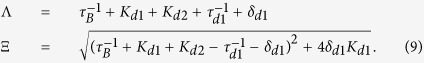


The LT spectrum 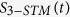 is the number of positrons annihilated in various times and is obtained as a negative time derivative of the decay spectrum 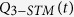



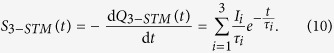


Hence the lifetimes 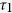, 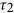, 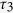 of the exponential components resolved in experimental LT spectra and their relative intensities 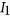, 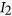, 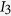 can be interpreted within 3-STM using Eqs (8. Note that for a high concentration of shallow or deep positron traps the lifetime 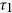 becomes extremely short and its intensity 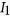 diminishes. In such case it is not possible to resolve the first component in LT spectrum due to limited resolution of LT spectrometer. This situation happened here in the case of low temperature LT spectra for CdTe:Cl. The short lived component could not be resolved in LT spectra due to very high density of shallow traps in this sample as will be shown in the following text.

The probability that a positron trapped in a shallow trap will escape by thermal excitation decreases with falling temperature. Hence the positron de-trapping rate from shallow traps 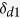 depends on temperature^53^


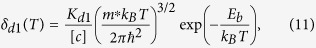


where 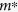 is the effective positron mass^26^, 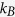 the Boltzmann constant, [*c*] the concentration of shallow traps, and 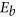 the positron binding energy to the shallow traps. The temperature dependence of the mean lifetime 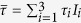 was fitted using 3-STM with 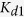, 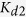, [*c*] and 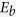 as fitting parameters. If the sample contains shallow traps, then the mean positron lifetime 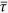 varies with temperature due to the temperature dependence of 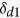. Figure 2 shows that 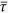 for CdTe:Cl decreases with temperature, which testifies to the presence of shallow traps in this sample. The temperature dependence of 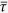 can be explained by the formation of series of attractive shallow Rydberg states connected with negatively-charged defects^54^,^55^. Since the positron binding energy of the shallow Rydberg states is rather small (typically ≤0.1 eV), they act as efficient positron traps at low temperatures only where the positron may not escape by thermal excitation^54^.

Hence, to describe LT spectra measured at RT 3-STM can be simplified by elimination of the shallow traps, i.e. by setting 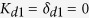, 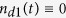 in Eqs (6. This leads to the following rate equations


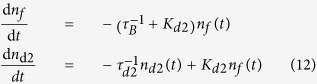


This simplified trapping model called two-state simple trapping model (2-STM) was used for analysis of the LT spectra measured at RT. The solution of 2-STM with the initial conditions 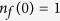 and 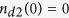 is two-component decay spectrum of positrons


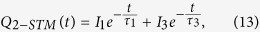


where


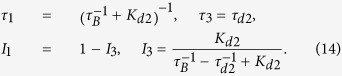


The LT spectrum 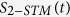 predicted by 2-STM is


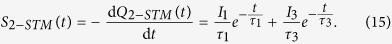


Decomposition of LT spectrum of the as-grown CdTe:Cl measured at RT revealed two exponential components, see Table 6. The shorter component with lifetime 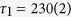 ps represents a contribution of free positrons. Note that 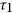 is shorter than the bulk positron lifetime for CdTe due to parallel positron annihilation and trapping in the deep traps, c.f. Eq. (14). The lifetime 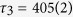 ps, 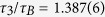, of the longer component is significantly higher than the positron lifetime for single vacancies in CdTe, see Table 3. Hence, we deduce that positrons in the as-grown CdTe:Cl are trapped in larger point defects with the open volume comparable with that of several vacancies. The positron trapping rate to these vacancy clusters can be calculated within 2-STM from Eqs. (14)


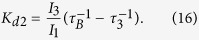


The concentration of vacancy clusters is directly proportional to the trapping rate 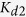 determined by 2-STM, 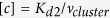. The specific positron trapping rate for the vacancy clusters was estimated as 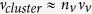, where 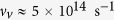 is the specific positron trapping rate for neutral vacancies in semiconductors^24^ and 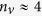 is the average number of vacancies constituting the cluster. The latter value was estimated by comparison of the ratio 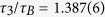 determined experimentally with the theoretical calculations of clusters of the neutral 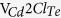 complexes of various sizes in Table 5.

Extended investigations of CdTe:Cl with chlorine content from 100 to 3000 ppm performed by Krause-Rehberg *et al*.^13^ revealed two components in LT spectra measured at RT: (i) the shorter component, with a lifetime of 330(10) ps, 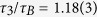, which was attributed to 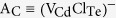 and (ii) a long-lived component with a lifetime of 450(15) ps, 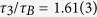, which was assigned to clusters of 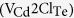 neutral complexes. The concentration of both defects increased with Cl content, testifying that they are associated with chlorine. Our CdTe:Cl sample exhibits a smaller lifetime 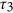 than 450 ps. This could be due to significantly lower Cl concentration leading to the formation of 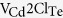 clusters of smaller size.

The analysis of the LT spectrum of the as-grown CdTe:Cl measured at 123 K revealed a new component with lifetime 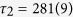 ps, which is shorter than the bulk positron lifetime (290 ps) and dominates the spectrum with 94% intensity, see Table 4. Taking into account the temperature dependence of 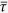 in Fig. 2, this component obviously comes from positrons trapped in the shallow Rydberg states associated with negatively-charged *A*-centres 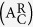. Indeed, the positron binding energy 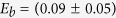 eV determined from fitting of the temperature dependence of 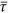 falls into the range 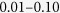 eV expected for the Rydberg states^52^. Evidently, 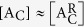. The concentration 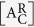 obtained from fitting of LT data by 3-STM is presented in Table 7. From the comparison of 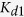 with 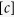 we can estimate the specific positron trapping rate for the shallow traps 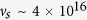 s^−1^. This value falls into the expected range for 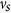 calculated in ref. 54.

Note that Rydberg states are characterized by a lifetime which is close to the bulk lifetime^54^,^55^. Depending on the local electron density the lifetime of positrons confined at low temperatures in shallow traps can be either higher or lower than the bulk positron lifetime. The latter case has been recently reported for Cu in-diffused GaAs:Te crystals^56^. Low temperature LT studies of Cu in-diffused GaAs:Te revealed shallow traps characterized by a lifetime of 220 ps, which is about of 8 ps shorter than the GaAs bulk lifetime of 228 ps^56^. The shallow traps in Cu-indiffused GaAs:Te were identified as 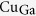 ions coupled with Ga vacancies 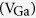. At RT positrons in the Cu-indiffused GaAs:Te sample are trapped at deep traps identified as 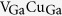 complexes and characterized by a positron lifetime of 280 ps. At low temperatures positrons are confined predominantly in Rydberg states associated with 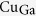 ions and characterized by a positron lifetime of 220 ps, which is shorter than the bulk lifetime^56^. Thus, it seems that positron trapping in the CdTe:Cl sample is to some extent analogous to that in Cu-indiffused GaAs:Te: at RT positrons are trapped at deep traps (vacancy clusters) while at low temperatures positrons are confined at Rydber states associated with Coulomb field around negatively charged A-centers and characterized by a positrons lifetime 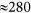 ps, which is about of 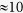 ps shorter than the CdTe bulk lifetime of 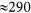 ps measured on the u-CdTe sample, see Table 4. We assume that the reduced lifetime of the positron in the Rydberg state comparing to the bulk lifetime may stem from a lattice relaxation and enhanced mass density in the vicinity of the vacancy, at the radius about 1 nm, where the positrons in Rydberg state mostly occur.

Annealing the CdTe:Cl sample in Cd vapour led to a significant reduction of the intensity of the long-lived defect component and shortening of its lifetime down to 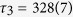 ps at RT. The ratio 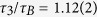 is close to the value calculated for 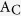 or single 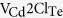 complex The reduction of this component is clearly caused by the removal of 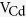 by Cd annealing and the reduction in cluster size. Ensuing analysis within 2-STM proves the depression of the density of 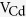 related defects, see Table 7. The Cd-annealed CdTe:Cl is characterized by very low resistivity and *n*-type conductivity, caused by 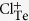 shallow donors which in contrast to the as-grown sample are not compensated by 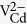 shallow acceptors. In spite of significant 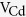 depreciation the sample measured at low temperature again exhibits a remarkable contribution of 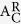, see Table 6. The temperature dependence of 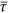 plotted in Fig. 2 is similar to the as-grown sample just shifted to lower values due to the suppressed contribution from neutral clusters. Since the lifetime of positrons localized in the shallow traps is lower than the CdTe bulk lifetime, 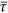 decreases below the bulk lifetime value at low temperatures where almost all positrons are confined in the shallow traps. Referring to similar concentration of defects at RT and at low temperature, see Table 7, we deduce that 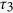 in the Cd-annealed CdTe:Cl sample is associated with 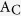 only.

Subsequent annealing of a CdTe:Cl sample in Te vapour leads to the appearance of a defect component with lifetime 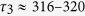 ps, which corresponds to negatively-charged 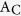 and neutral 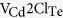 defects. Unlike the as-grown CdTe:Cl crystal, the 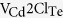 complexes formed after Te-annealing are isolated and do not agglomerate into clusters. This could be caused by the fact that during Te-annealing the sample was kept at a lower temperature than during crystal growth, and the mobility of the 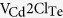 complexes was therefore lower. The analysis of LT spectra measured at 123 K revealed that the positron trapping in 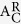 dominates at low temperatures in a similar way as in the Cd-annealed crystal. The temperature dependence of 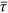 presented in Fig. 2 again shows a drop with decreasing temperature in a similar manner as for the Cd-annealed sample.

Referring to the concentration of defects (Table 7), we can deduce that the defects observed at RT in the Te-annealed sample are associated with 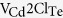 and 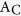 at the total density 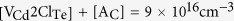. Similarly, the low temperature measurements allow us to establish 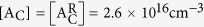, which yields 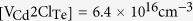. Consequently, the calculation of the total Cl density in 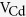-related complexes provides 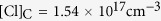. This result is very close to the concentration of chlorine determined by GDMS 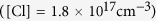. Since GDMS analysis is affected by up to 30% error, we consider this finding as an excellent confirmation of our assignment of defects.

Similar estimation can be done also for the as-grown CdTe:Cl sample where the concentration of 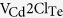 clusters of 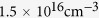 was estimated in Table 7. Assuming that each cluster consists on average of 4 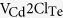 complexes (as deduced from the ratio 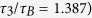 the concentration of Cl contained in 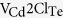 clusters is 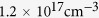. At the same time the low temperature measurement of the same sample yielded 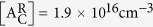. Hence, the total concentration of Cl in 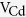-related complexes in the as-grown CdTe:Cl is 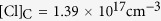. Again this value is in very reasonable agreement with the total Cl density determined by GDMS.

The as-grown CdTe:Cl crystal was characterized also by positron back-diffusion measurement on the slow positron beam. The dependence of the *S*-parameter on the energy *E* of incident positrons for the as-grown CdTe:Cl crystal is plotted in Fig. 3. Obviously the 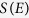 curve for the as-grown CdTe:Cl sample exhibits similar features as the curve for the u-CdTe crystal, namely a maximum at low energies (≈2 keV) followed by a gradual decrease down to a plateau value at high energies where virtually all positrons are annihilated inside the CdTe:Cl region. However the plateau value at high energies for the as-grown CdTe:Cl sample is remarkably higher than that for the u-CdTe. The 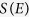 curve was fitted in the similar manner as the curve for u-CdTe sample, i.e. using a two-layer model consisting of a thin surface oxide layer and the bulk CdTe:Cl region. The model curve calculated by VEPFIT^25^ is plotted by solid line in Fig. 3 and describes the experimental points accurately. The thickness of the oxide layer of ≈20 nm obtained from fitting is comparable with that measured in the u-CdTe. The positron diffusion length in the CdTe:Cl region is 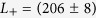 nm. This value is shorter than the positron diffusion length 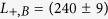 nm determined for the u-CdTe. Shortened positron diffusion length and higher S parameters at high energies give clear evidence that the as-grown CdTe:Cl sample exhibits higher density of defects than the u-CdTe. The concentration of defects [*c*] in the as-grown CdTe:Cl sample can be estimated from shortening of the positron diffusion length using the expression^57^


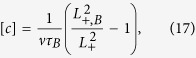


where *v* is the specific positron trapping rate to defects. From LT studies we know that the as-grown CdTe:Cl sample contains 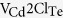 clusters consisting on average of four 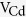. Assuming 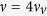 one gets 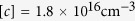 which is in very reasonable agreement with the concentration of 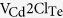 clusters 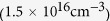 determined by LT spectroscopy in Table 7.

### CdZnTe:Ge

At RT the CdZnTe:Ge as-grown crystal exhibits a defect component with lifetime 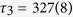 ps falling into the range 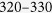 ps commonly attributed to 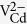 shallow acceptors or complexes of 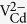 associated with donors, see Table 4. The lifetime 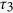 at 123 K is practically the same as in RT, and 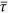 remains constant apart from the statistical scattering in the whole temperature range of 123–295 K, see Fig. 2. A similar result was observed^17^ for undoped CdZnTe. The temperature independence of 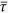 observed in CdZnTe:Ge crystal testifies that (i) the defects responsible for the lifetime 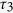 in this sample are mostly electrically neutral, and (ii) unlike CdTe:Cl, the CdZnTe:Ge crystal does not contain shallow traps in detectable concentration.

Taking into account electrical neutrality of defects and positron lifetime comparable to 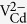 the component with the lifetime 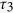 can be attributed to positrons trapped at electrically neutral 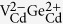 complexes which are likely formed in CdZnTe:Ge. Table 7 shows the concentration of 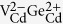 complexes determined as 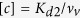, where 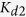 is the trapping rate calculated within 2-STM by Eq. (16) and 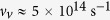 is the specific positron trapping rate for neutral vacancies in semiconductors^24^.

Annealing of CdZnTe:Ge crystal in Cd vapour led to the disappearance of the defect component with lifetime 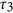, and the LT spectrum became single-component with the lifetime 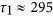 ps, which is close to the bulk lifetime of 292(1) ps measured in the u-CdTe, see Table 6. Hence, virtually all positrons in the Cd-annealed CdZnTe:Ge sample are annihilated in the de-localized state. This result proves that 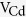 annihilation is due to Cd-annealing. Slightly higher value of the bulk positron for CdZnTe:Ge compared to that for u-CdTe could be caused by lower electron density in CdZnTe compared to that in CdTe. Temperature-dependent measurements did not reveal any temperature dependence of LT results for the Cd-annealed CdZnTe:Ge sample, see Fig. 2 and Table 6, which is in accordance with the assignment of the 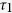 component to the free positrons.

The subsequent annealing of CdZnTe:Ge in Te vapour resulted in the restoration of the defect component with lifetime 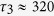 ps, which shows the re-emergence of 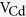. The LTs obtained after annealing are very similar to those measured in the as-grown CdZnTe:Ge, including the temperature independence of 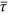 in Fig. 2, which testifies to positron trapping in electrically neutral 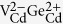 complexes.

### CDB spectroscopy

**Figure 4:**
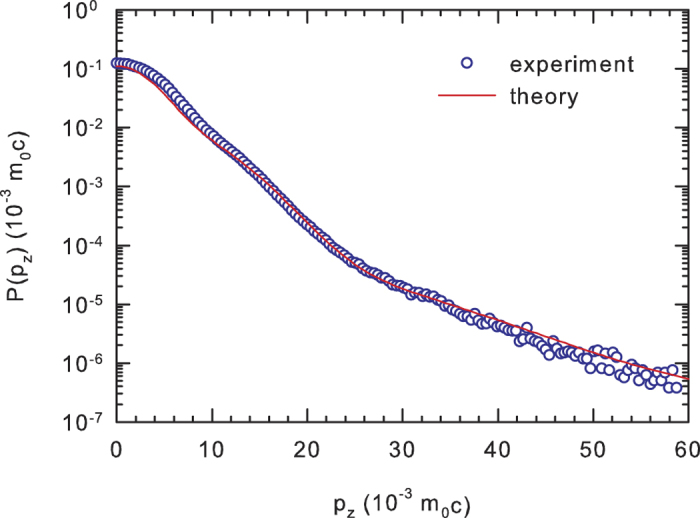
The positron annihilation momentum density *P*(*p*_*z*_) measured by CDB in the undoped CdTe sample and calculated for a perfect CdTe crystal using the ATSUP-GGA approach.

The CDB spectroscopy was employed in order to obtain direct information about chemical environment of 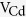 in CdTe:Cl and CdZnTe:Ge crystals. Figure 4 shows the momentum distribution of annihilating electron-positron pairs measured in the u-CdTe reference sample. The calculated momentum distribution for a perfect CdTe crystal is plotted in the figure as well. One can see in the figure that the momentum distribution calculated using the ATSUP-GGA approach agrees well with the experimental points in the high momentum range 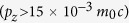 where the contribution of positrons annihilated by core electrons dominates. This is caused by the fact that core electrons in the inner shells retain their atomic character are almost not influenced by crystal bonding. On the other hand the ATSUP-based approach is not able to describe accurately the momentum distribution in the the low momentum region 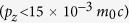 where the contribution of positrons annihilated by valence electrons becomes dominating. For this reason the calculated momentum distributions will be compared with the experimental data in the high momentum region only.

**Figure 5:**
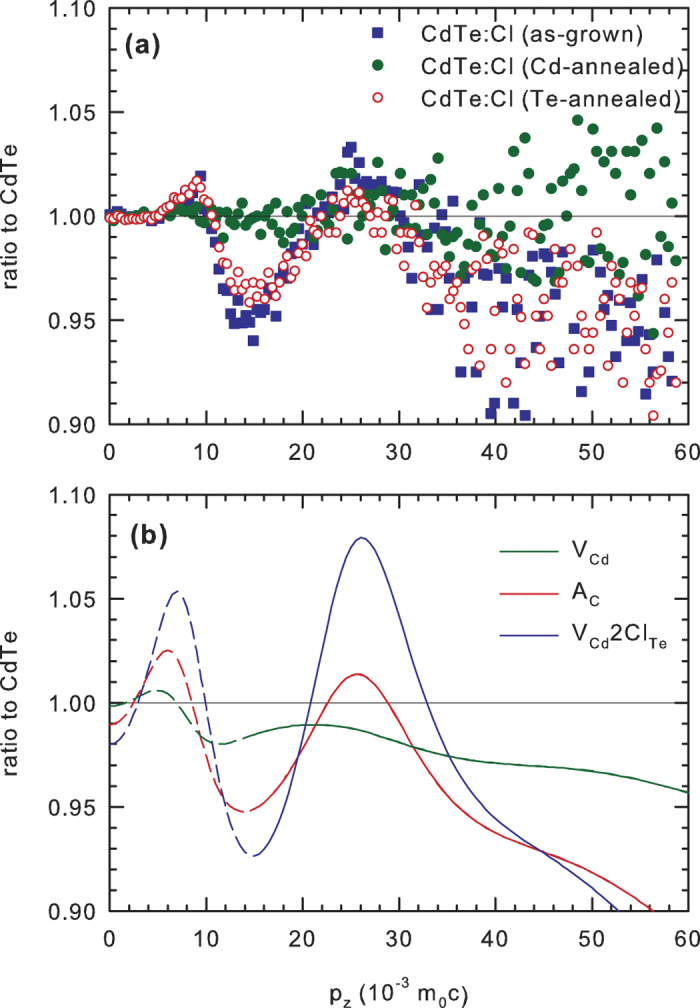
The CDB ratio curves (related to defect-free u-CdTe) (**a**) experimental data for CdTe:Cl in the as-grown state and after annealing in Cd and Te vapour; (**b**) calculated ratio curves for V_*Cd*_, A_*C*_ and V_*Cd*_2*Cl*_*Te*_ defects. The calculated curves are plotted by dashed lines in the low momentum range (*p*_*z*_ < 15 × 10^−3^*m*_0_*c*) where the accuracy of the momentum distribution calculated by ASTUP is poor.

Figure 5a shows the experimental CDB ratio curves related to the u-CdTe reference for the CdTe:Cl sample in the as-grown state and after annealing in Cd and Te vapour, respectively. The calculated CDB curves related to a perfect CdTe crystal for 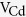, 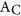 and 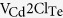 defects are plotted in Fig. 5b. From inspection of Fig. 5 it becomes clear that the CDB curve for CdTe:Cl annealed in Cd vapour is almost flat (except of statistical scattering) and close to unity in the whole momentum range testifying that the momentum distribution for the Cd-annealed CdTe:Cl is very similar to that in the u-CdTe reference. This confirms the picture that positron traps were removed by annealing in Cd vapour and virtually all positrons in the Cd-annealed sample are annihilated in the free state. On the other hand, the CDB ratio curves for the as-grown CdTe:Cl and the sample annealed in Te vapour clearly differ from unity and exhibit a distinct peak in the high momentum range at 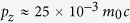. Similar peak is observed in the CDB curves calculated for positrons trapped in 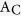 and 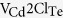 defects in Fig. 5b. This testifies to positron trapping in defects associated with chlorine in the as-grown and Te-annealed CdTe:Cl sample.

**Figure 6:**
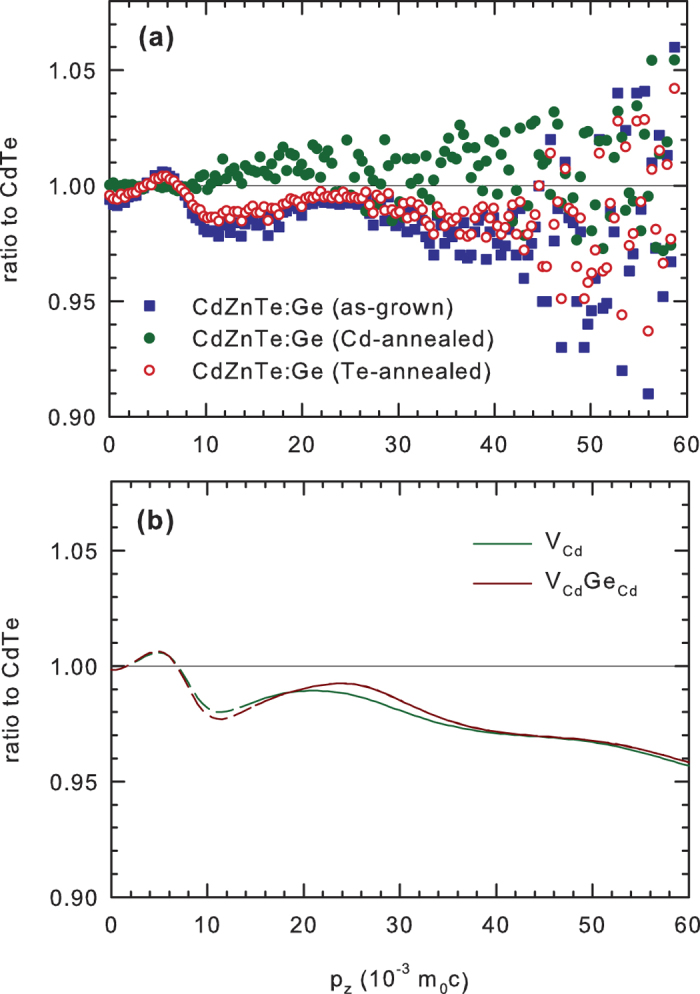
The CDB ratio curves (related to defect-free u-CdTe) (**a**) experimental data for CdZnTe:Ge in the as-grown state and after annealing in Cd and Te vapour; (**b**) calculated ratio curves for V_Cd_ and V_Cd_Ge_Cd_ defects. The calculated curves are plotted by dashed lines in the low momentum range (*p*_*z*_ < 15 × 10^−3^ *m*_0_*c*) where the accuracy of the momentum distribution calculated by ASTUP is poor.

The CDB results for CdZnTe:Ge samples are presented in Fig. 6. The experimental CDB curves for the as-grown and Cd or Te-annealed CdZnTe:Ge are plotted in Fig. 6a while Fig. 6b shows the CDB ratio curves calculated for positrons trapped at 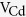 and 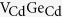 defects. The CDB ratio curve for Cd-annealed CdZnTe:Ge sample is again almost flat and close to unity indicating that the momentum distribution in this sample is similar to that in the u-CdTe reference. A slight enhancement in the momentum range 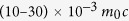 is most probably due to positrons annihilated by electrons belonging to Zn. Annealing in Te vapour modified the momentum distribution of CdZnTe:Ge sample. Shape of the ratio curve for the Te-annealed sample is very similar to the curves calculated for positrons trapped at 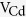 and 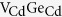 defects. Hence, in accordance with the results of LT spectroscopy the CDB data in Fig. 6 indicate that positrons in CdZnTe:Ge annealed in Te vapour are trapped at defects containing 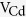. Very similar CDB curve was observed also in the as-grown sample. However, CDB spectroscopy does not allow to discern positrons trapped in isolated 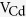 from those trapped in 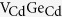 complexes due to very similar CDB ratio curves for both defects, see Fig. 6b.

In spite of extensive research, the basic properties of native point defects in CdTe/CdZnTe are still far from agreed understanding. Both formation and ionization energies are not settled and analyses based on various defect models or experimental data produce incompatible defect characteristics^8^. Nowadays, it is generally assumed that the difficulties in detecting of native point defects ensue from their reactivity and fast diffusion at elevated temperature. During cooling to room temperature native defects are supposed to migrate and interact with charged impurities. As a result, various types of associates and precipitates are formed^58^,^59^. The remaining isolated 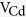 mostly annihilate during cooling at surface or dislocations and the final room temperature density of 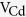 comes out very low^42^. Evidently, such premise is in pure agreement with evaluated defect structure both in CdTe:Cl and CdZnTe:Ge. Analysis of PAS data suggests that all detected 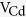 are incorporated in various complexes stabilizing them in the lattice.

Detailed characterization of defect structure of CdTe:Cl and CdZnTe:Ge samples summarized in Table 7 raises a query on the unambiguity of the presented defect model. We believe that the comprehensive exploration of CdTe:Cl and CdZnTe:Ge samples, which were studied both in Cd- and in Te-rich state by multiple techniques, LT, CDB and galvanomagnetic measurements, and characterized by GDMS, allowed us the interpreting of measured data by rather unique way. In view of the fact that the defect densities in Te-rich state significantly exceed the densities of obvious residual extrinsic acceptor, we may conclude that 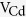 is the only candidate for the acceptor defect at these conditions.

Similarly, the intentional doping by Cl or Ge donors supplied the only extrinsic elements with the large enough concentration to arrange the detected density of revealed A-centres and complexes. Nevertheless, suggested interpretation of the defect structure of complexes does not represent the only possibility to create neutral or acceptor-type complex in CdTe and CdZnTe. An analogous defect might be created also without participation of extrinsic atoms taking only native point defects into account. That is the complex of Cd vacancy and Te anti-site defect 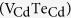 predicted in ref. 60. Such complex was not identified in experiments yet and its electrical properties are not known. The reason, why we favour the structure of complex related to extrinsic Cl or Ge donors rises from the proximity of the evaluated complex densities in Te-annealed samples and respective element concentrations revealed by GDMS. The density of 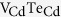 complexes appears significantly below extrinsic-donor-related complexes and does not manifest in presented measurements. This picture is supported also by theoretical calculations in Table 3 since the 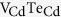 complex is characterized positron lifetime which is slightly lower that the lifetime for isolated 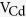. But such trend was not observed in experimental lifetimes.

## Conclusions

Point defects in CdTe:Cl and CdZnTe:Ge crystals have been characterized by LT and CDB spectroscopies combined with galvanomagnetic measurements. The as-grown CdTe:Cl crystal exhibits larger point defects with open volume comparable with several vacancies. The low temperature LT studies in CdTe:Cl have proved the existence of negatively-charged shallow traps interpreted as Rydberg states associated with Cl-related *A*-centres. These shallow traps are able to confine positrons at low temperatures only when thermal de-trapping becomes sufficiently small. The lifetime of positrons localized in the shallow traps is shorter than the CdTe bulk lifetime. The as-grown CdZnTe:Ge crystal contains 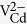 shallow acceptors forming neutral complexes with 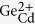 deep donors. It has been confirmed that Cd-rich annealing of both CdTe:Cl and CdZnTe:Ge crystals suppresses the concentration of 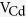, which are created again during subsequent Te-rich annealing.

## Additional Information

**How to cite this article**: Šedivý, L. *et al*. Positron annihilation spectroscopy of vacancy-related defects in CdTe:Cl and CdZnTe:Ge at different stoichiometry deviations. *Sci. Rep.* **6**, 20641; doi: 10.1038/srep20641 (2016).

## Figures and Tables

**Table 1:** The concentrations of elements exceeding 10^15^ cm^−3^ for u-CdTe, CdTe:Cl and CdZnTe:Ge established by GDMS in units 10^15^ cm^−3^.

Sample	B	Mg	Al	Si	P	S	Cl	Ca	Au	Cr	Ni	Cu	Zn	Ge	Se	Ag	In	Sn
u-CdTe							1						2		2	2	1	1
CdTe:Cl		1	1	1		5	183		1			3	3					
CdZnTe:Ge	3	5	3	3	1	8		1	7	20	3	1	3.5%	60	12			

A 30% error of GDMS analysis was declared.

**Table 2:** Summary of galvanomagnetic measurements: resistivity of samples, type of conductivity and concentration of free carriers.

sample	treatment	resistivity(Ω · *cm*)	type	concentration(*cm*^−3^)
u-CdTe	as-grown	5.5	*n*	1.1 × 10^15^
CdTe:Cl	as-grown	7.3 × 10^7^	*p*	1.5 × 10^10^
	Cd-annealed	4.9 × 10^−2^	*n*	9.7 × 10^16^
	Te-annealed	6.9 × 10^3^	*p*	8.5 × 10^12^
CdZnTe:Ge	as-grown	1.8 × 10^9^	*n*	6.1 × 10^6^
Cd-annealed	3.0 × 10^4^	*p*	3.2 × 10^12^	
Te-annealed	2.5 × 10^3^	*p*	4.0 × 10^13^	

**Table 3:** Theoretical positron lifetimes for a perfect CdTe crystal and various defects calculated by various approaches.

positron state	ATSUP	ATSUP	VASP	ATSUP	ATSUP	ATSUP
	-LDA	-GGA	-GGA	-LDA	-LDA	-GGA
				Ref. 15	Ref. 45	Ref. 15
	(ps)	(ps)	(ps)	(ps)	(ps)	(ps)
bulk	276	307	291	276	276	309
V_Cd_	289	323	325	291	288	322
V_Cd_Te_Cd_	287	315				
V_Cd_Ge_Cd_	290	323				
A_C_	306	331	330			
V_Cd_2Cl_Te_	316	346				
(V_Cd_2Cl_Te_)_2_ cluster	319	365				
(V_Cd_2Cl_Te_)_3_ cluster	338	401				
(V_Cd_2Cl_Te_)_4_ cluster	351	427				

Theoretical lifetimes available in literature are shown in the table as well.

**Table 4:** Experimental positron lifetimes for a perfect CdTe crystal and selected defects reported in literature.

positron state	lifetime(ps)	*τD*/*τB*	reference	note
bulk	280(1)		13	
	283(1)		46	
	285(1)		16,18	In-doped
	287(1)		14	
	290(4)		15	CdTe film
	291(1)		47	
V_Cd_	325(5)	1.15(2)	18,46	electron irradiated
	320(2)	1.123(7)	16,18	In-doped
	323(3)	1.13(1)	14	In-doped
	321(9)	1.11(3)	15	CdTe film
A_C_	330(10)	1.18(3)	13	Cl-doped

The *τ*_*D*_/*τ*_*B*_ ratio is shown in the table for positrons trapped at defects. Note that in the ratios the lifetime of trapped positrons was always related to the bulk lifetime measured on the same setup, i.e. *τ*_*D*_ values were divided by *τ*_*B*_ ones published by the same authors. The uncertainties (one standard deviations) are given in parentheses in the units of the last significant digit.

**Table 5:** Theoretical *τ*_*D*_/*τ*_*B*_ ratios for positrons trapped at various defects in CdTe calculated by various approaches.

positron state	ATSUP	ATSUP	VASP	ATSUP	ATSUP	ATSUP
	-LDA	-GGA	-GGA	-LDA	-LDA	-GGA
				Ref. 15	Ref. 45	Ref. 15
V_Cd_	1.05	1.05	1.12	1.05	1.04	1.04
V_Cd_Te_Cd_	1.04	1.03				
V_Cd_Ge_Cd_	1.05	1.05				
A_C_	1.11	1.08	1.13			
V_Cd_2Cl_Te_	1.14	1.13				
(V_Cd_2Cl_Te_)_2_ cluster	1.16	1.19				
(V_Cd_2Cl_Te_)_3_ cluster	1.22	1.31				
(V_Cd_2Cl_Te_)_4_ cluster	1.27	1.39				

Theoretical *τ*_*D*_/*τ*_*B*_ ratios available in literature are shown in the table as well.

**Table 6:** Results of LT studies: lifetime *τ*_*i*_ and the relative intensity *I*_*i*_ of the exponential components resolved in the LT spectra evaluated at 295 K and 123 K.

sample	treatment	T (K)	*τ*_1_ (ps)	I_1_ (%)	*τ*_2_ (ps)	I_2_ (%)	*τ*_3_ (ps)	I_3_ (%)
u-CdTe	as-grown	295	292(1)	100	—	—	—	—
		123	290(1)	100	—	—	—	—
CdTe:Cl	as-grown	295	230(2)	45(1)	—	—	405(2)	55(1)
		123	—	—	281(9)	94(5)	400(10)	6(2)
	Cd-annealed	295	283(8)	81(5)	—	—	328(7)	19(5)
		123	—	—	280(10)	98(5)	330(10)	2(1)
	Te-annealed	295	240(10)	22(5)	—	—	316(6)	78(5)
		123	—	—	278(8)	93(5)	320(10)	7(3)
CdZnTe:Ge	as-grown	295	263(9)	42(1)	—	—	327(8)	58(1)
		123	260(9)	37(4)	—	—	326(8)	63(1)
	Cd-annealed	295	295.0(4)	100	—	—	—	—
		123	293.0(5)	100	—	—	—	—
	Te-annealed	295	260(10)	40(5)	—	—	320(5)	60(10)
		123	253(8)	45(5)	—	—	325(5)	55(8)

The uncertainties (one standard deviations) are given in parentheses in the units of the last significant digit.

**Table 7:** The concentration of defects calculated from the LT data.

sample	treatment	defect type	[c] (cm^−3^)
u-CdTe	as-grown	—	<1 × 10^15^
CdTe:Cl	as-grown	V_Cd_2Cl_Te_ cluster	1.5(1) × 10^16^
		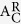	1.9(3) × 10^16^
	Cd-annealed	A_C_	5(1) × 10^15^
		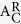	7(2) × 10^15^
	Te-annealed	A_C_ and V_Cd_2Cl_Te_	9.0(5) × 10^16^
		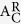	2.6(6) × 10^16^
CdZnTe:Ge	as-grown	V_Cd_Ge_Cd_	2.6(2) × 10^16^
	Cd-annealed	—	<1 × 10^15^
	Te-annealed	V_Cd_Ge_Cd_	2.7(2) × 10^16^

The neutral defects at CdTe:Cl and at CdZnTe:Ge, negatively-charged *A*-centres and shallow Rydberg states associated with negatively-charged *A*-centres are symbolized by V_Cd_2Cl_Te_, V_Cd_Ge_Cd_, A_C_, 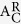, respectively.

## References

[b1] Fang Z., Wang X. C., Wu H. C. & Zhao C. Z. Achievements and challenges of CdS/CdTe solar cells. Int. J. Photoenergy 2011, 8 (2011).

[b2] Kranz L., Buecheler S. & Tiwari A. N. Technological status of CdTe photovoltaics. Sol. Energ. Mat. Sol. Cells 119, 278–280 (2013).

[b3] Gessert T. A. . Research strategies toward improving thin-film CdTe photovoltaic devices beyond 20% conversion efficiency. Sol. Energ. Mat. Sol. Cells 119, 149–155 (2013).

[b4] Szeles C. CdZnTe and CdTe materials for X-ray and gamma ray radiation detector applications. Phys. status solidi (b) 241, 783–790 (2004).

[b5] del Sordo S. . Progress in the development of CdTe and CdZnTe semiconductor radiation detectors for astrophysical and medical applications. Sensors 9, 3491–3526 (2009). 22412323 10.3390/s90503491 PMC3297127

[b6] Nishimura T., Aritome H., Masuda K. & Namba S. Optical Waveguiding and Electrooptic Modulation in Ion-Implanted CdTe. Jpn. J. Appl. Phys. 15, 2283–2284 (1976).

[b7] Sen S., Liang C. S., Rhiger D. R., Stannard J. E. & Arlinghaus H. F. Reduction of CdZnTe substrate defects and relation to epitaxial HgCdTe quality. J. Electron. Mater. 25, 1188–1195 (1996).

[b8] Schlesinger T. . Cadmium zinc telluride and its use as a nuclear radiation detector material. Mater. Sci. Eng. R-Rep. 32, 103–189 (2001).

[b9] Franc J. . Melt growth and post-grown annealing of semiinsulating (CdZn)Te by vertical gradient freeze method. Cryst. Res. Technol. 48, 214–220 (2013).

[b10] Carvalho A., Tagantsev A. K., Öberg S., Briddon P. R. & Setter N. Cation-site intrinsic defects in Zn-doped CdTe. Phys. Rev. B 81, 75215 (2010).

[b11] Rudolph P. Non-stoichiometry related defects at the melt growth of semiconductor compound crystals a review. Cryst. Res. Technol. 38, 542–554 (2003).

[b12] Biswas K. & Du M.-H. What causes high resistivity in CdTe. New J. Phys. 14, 63020 (2012).

[b13] Krause-Rehberg R., Leipner H., Abgarjan T. & Polity A. Review of defect investigations by means of positron annihilation in II-VI compound semiconductors. Appl. Phys. A Mater. Sci. Process. 66, 599–614 (1998).

[b14] Kauppinen H., Baroux L., Saarinen K., Corbel C. & Hautojärvi P. Identification of cadmium vacancy complexes in CdTe(In), CdTe(Cl) and CdTe(I) by positron annihilation with core electrons. J. Phys. Condens. Matter 9, 5495–5505 (1997).

[b15] Keeble D. J., Major J. D., Ravelli L., Egger W. & Durose K. Vacancy defects in CdTe thin films. Phys. Rev. B 84, 174122 (2011).

[b16] Gély-Sykes C., Corbel C. & Triboulet R. Positron trapping in vacancies in indium doped CdTe crystals. Solid State Commun. 80, 79–83 (1991).

[b17] Martyniuk M. & Mascher P. Investigation of the defect structure in Cd1âˆ’xZnxTe by positron lifetime spectroscopy. Phys. B Condens. Matter 308–310, 924–927 (2001).

[b18] Corbel C., Baroux L., Kiessling F., Gély-Sykes C. & Triboulet R. Positron trapping at native vacancies in CdTe crystals: In doping effect. Mater. Sci. Eng. B 16, 134–138 (1993).

[b19] Boerger D. M. Generalized Hall-effect measurement geometries and limitations of van der Pauw-type Hall-effect measurements. J. Appl. Phys. 52, 269 (1981).

[b20] Höschl P. . Electrical and luminescence properties of (CdZn)Te single crystals prepared by the vertical gradient freezing method. J. Cryst. Growth 184–185, 1039–1043 (1998).

[b21] Bečvář F., Čížek J., Procházka I. & Janotová J. The asset of ultra-fast digitizers for positron-lifetime spectroscopy. Nucl. Instrum. Meth. A 539, 372–385 (2005).

[b22] Asoka-Kumar P. . Increased Elemental Specificity of Positron Annihilation Spectra. Phys. Rev. Lett. 77, 2097–2100 (1996). 10061856 10.1103/PhysRevLett.77.2097

[b23] Čížek J., Vlček M. & Procházka I. Digital spectrometer for coincidence measurement of Doppler broadening of positron annihilation radiation. Nucl. Instrum. Meth. A 623, 982–994 (2010).

[b24] Krause-Rehberg R. & Leipner H. S. Positron Annihilation in Semiconductors, vol. 127 of Springer Series in Solid-State Sciences (Springer-Verlag, Berlin, 1999), 1 edn.

[b25] van Veen A. . VEPFIT applied to depth profiling problems. Appl. Surf. Sci. 85, 216–224 (1995).

[b26] Puska M. J. & Nieminen R. M. Theory of positrons in solids and on solid surfaces. Rev. Mod. Phys. 66, 841–897 (1994).

[b27] Boroński E. & Nieminen R. Electron-positron density-functional theory. Phys. Rev. B 34, 3820–3831 (1986). 10.1103/physrevb.34.3820 9940145

[b28] Puska M., Mäkinen S., Manninen M. & Nieminen R. Screening of positrons in semiconductors and insulators. Phys. Rev. B 39, 7666–7679 (1989). 10.1103/physrevb.39.7666 9947448

[b29] Perkowitz S. & Thorland R. Far-infrared study of free carriers and the plasmon-phonon interaction in CdTe. Phys. Rev. B 9, 545–550 (1974).

[b30] Barbiellini B. . Calculation of positron states and annihilation in solids: A density-gradient-correction scheme. Phys. Rev. B 53, 16201–16213 (1996). 10.1103/physrevb.53.16201 9983453

[b31] Desclaux J. A multiconfiguration relativistic DIRAC-FOCK program. Comput. Phys. Commun. 9, 31–45 (1975).

[b32] Puska M. J. & Nieminen R. M. Defect spectroscopy with positrons: a general calculational method. J. Phys. F Met. Phys. 13, 333–346 (1983).

[b33] Korhonen T., Puska M. & Nieminen R. First-principles calculation of positron annihilation characteristics at metal vacancies. Phys. Rev. B 54, 15016–15024 (1996). 10.1103/physrevb.54.15016 9985557

[b34] Puska M., Seitsonen A. & Nieminen R. Electron-positron Car-Parrinello methods: Self-consistent treatment of charge densities and ionic relaxations. Phys. Rev. B 52, 10947–10961 (1995). 10.1103/physrevb.52.10947 9980193

[b35] Campillo Robles J. M., Ogando E. & Plazaola F. Positron lifetime calculation for the elements of the periodic table. J. Phys. Condens. Matter 19, 176222 (2007). 21690967 10.1088/0953-8984/19/17/176222

[b36] Kresse G. & Hafner J. Ab initio molecular dynamics for liquid metals. Phys. Rev. B 47, 558–561 (1993). 10.1103/physrevb.47.558 10004490

[b37] Kresse G. Efficient iterative schemes for ab initio total-energy calculations using a plane-wave basis set. Phys. Rev. B 54, 11169–11186 (1996). 10.1103/physrevb.54.11169 9984901

[b38] Kresse G. From ultrasoft pseudopotentials to the projector augmented-wave method. Phys. Rev. B 59, 1758–1775 (1999).

[b39] Monkhorst H. J. & Pack J. D. Special points for Brillouin-zone integrations. Phys. Rev. B 13, 5188–5192 (1976).

[b40] Alatalo M. . Theoretical and experimental study of positron annihilation with core electrons in solids. Phys. Rev. B 54, 2397–2409 (1996). 10.1103/physrevb.54.2397 9986086

[b41] Kuriplach J., Morales A., Dauwe C., Segers D. & Šob M. Vacancies and vacancy-oxygen complexes in silicon: Positron annihilation with core electrons. Phys. Rev. B 58, 10475–10483 (1998).

[b42] Grill R. . Semi-insulating Te-saturated CdTe. IEEE Trans. Nucl. Sci. 52, 1925–1931 (2005).

[b43] Belas E. . Preparation of Semi-Insulating CdTe:In by Post-Grown Annealing After Elimination of Te Inclusions. IEEE Trans. Nucl. Sci. 54, 786–791 (2007).

[b44] Belas E. . Regular and anomalous-type conversion of p-CdTe during Cd-rich annealing. J. Electron. Mater. 34, 957–962 (2005).

[b45] Plazaola F., Seitsonen A. P. & Puska M. J. Positron annihilation in II-VI compound semiconductors: theory. J. Phys. Condens. Matter 6, 8809–8827 (1994).

[b46] Gély C., Corbel C. & Triboulet C. Positron lifetime measurements in a ii-vi-compound - indium doped cdte. Acad. Sci. Paris 309, 179–182 (1989).

[b47] Dannefaer S. A systematic study of positron lifetimes in semiconductors. J. Phys. C Solid State Phys. 15, 599–605 (1982).

[b48] Choi S. S. & Lucovsky G. Native oxide formation on CdTe. J. Vac. Sci. Technol. B 6, 1198 (1988).

[b49] Badano G., Million A., Canava B., Tran-Van P. & Etcheberry A. Fast Detection of Precipitates and Oxides on CdZnTe Surfaces by Spectroscopic Ellipsometry. J. Electron. Mater. 36, 1077–1084 (2007).

[b50] Neretina S. . Defect characterization of CdTe thin films using a slow positron beam. Phys. Stat. Sol. c 4, 3659 (2007).

[b51] Smedskjaer L. C., Manninen M. & Fluss M. J. An alternative interpretation of positron annihilation in dislocations. J. Phys. F Met. Phys. 10, 2237–2249 (1980).

[b52] Hautojärvi P. & Corbel C. Positron spectroscopy of defects in metals and semiconductors. In Dupasqier A. & Mills A. P. (eds.) Positron spectroscopy of solids, vol. 125 of Proc. Int. Sch. Phys. {‘Enrico Fermi’}, Course CXXV 491–532 (IOS Press, Amsterdam, 1995).

[b53] Manninen M. & Nieminen R. M. Positron detrapping from defects: A thermodynamic approach. Appl. Phys. A Mater. Sci. Process. 26, 93–100 (1981).

[b54] Puska M., Corbel C. & Nieminen R. Positron trapping in semiconductors. Phys. Rev. B 41, 9980–9993 (1990). 10.1103/physrevb.41.9980 9993382

[b55] Corbel C., Pierre F., Saarinen K., Hautojärvi P. & Moser P. Gallium vacancies and gallium antisites as acceptors in electron-irradiated semi-insulating GaAs. Phys. Rev. B 45, 3386–3399 (1992). 10.1103/physrevb.45.3386 10001913

[b56] Elsayed M., Krause-Rehberg R., Anwand W., Butterling M. & Korff B. Identification of defect properties by positron annihilation in Te-doped GaAs after Cu in-diffusion. Phys. Rev. B 84, 195208 (2011).

[b57] Veen A. v., Schut H., Vries J. d., Hakvoort R. A. & Ijpma M. R. Analysis of positron profiling data by means of vepfit. In Schultz, P., Massoumi, G. & Simpson, P. (eds.) *AIP Conference Proceedings No. *218, 83 (AIP, New York, 1990).

[b58] Marfaing Y. Self-compensation in ii-vi compounds. Prog. Cryst. Growth Charact. Mater. 4, 317–343 (1981).

[b59] Marfaing Y. Impurity compensation. In Siffert R. & Triboulet P. (eds) CdTe and Related Compounds; Physics, Defects, Hetero- and Nano-structures, Crystal Growth, Surfaces and Applications, European Materials Research Society Series. chap. VII, 363–388 (Elsevier, Amsterdam, 2010), first edition edn.

[b60] Berding M. A. Annealing conditions for intrinsic CdTe. Appl. Phys. Lett. 74, 552 (1999).

